# Progranulin reduces insoluble TDP-43 levels, slows down axonal degeneration and prolongs survival in mutant TDP-43 mice

**DOI:** 10.1186/s13024-018-0288-y

**Published:** 2018-10-16

**Authors:** Sander Beel, Sarah Herdewyn, Raheem Fazal, Mathias De Decker, Matthieu Moisse, Wim Robberecht, Ludo Van Den Bosch, Philip Van Damme

**Affiliations:** 1 Department of Neurosciences, Experimental Neurology and Leuven Brain Institute (LBI), KU Leuven - University of Leuven, Leuven, Belgium; 2 VIB, Center for Brain & Disease Research, Laboratory of Neurobiology, Leuven, Belgium; 3 Department of Neurology, University Hospital Leuven, Herestraat 49, 3000 Leuven, Belgium

**Keywords:** Frontotemporal dementia, Amyotrophic lateral sclerosis, TDP-43, Progranulin

## Abstract

**Background:**

TAR DNA binding protein 43 (TDP-43) is the main disease protein in most patients with amyotrophic lateral sclerosis (ALS) and about 50% of patients with frontotemporal dementia (FTD). TDP-43 pathology is not restricted to patients with missense mutations in *TARDBP*, the gene encoding TDP-43, but also occurs in ALS/FTD patients without known genetic cause or in patients with various other ALS/FTD gene mutations. Mutations in *progranulin* (*GRN*), which result in a reduction of ~ 50% of progranulin protein (PGRN) levels, cause FTD with TDP-43 pathology. How loss of PGRN leads to TDP-43 pathology and whether or not PGRN expression protects against TDP-43-induced neurodegeneration is not yet clear.

**Methods:**

We studied the effect of PGRN on the neurodegenerative phenotype in TDP-43(A315T) mice.

**Results:**

PGRN reduced the levels of insoluble TDP-43 and histology of the spinal cord revealed a protective effect of PGRN on the loss of large axon fibers in the lateral horn, the most severely affected fiber pool in this mouse model. Overexpression of PGRN significantly slowed down disease progression, extending the median survival by approximately 130 days. A transcriptome analysis did not point towards a single pathway affected by PGRN, but rather towards a pleiotropic effect on different pathways.

**Conclusion:**

Our findings reveal an important role of PGRN in attenuating mutant TDP-43-induced neurodegeneration.

**Electronic supplementary material:**

The online version of this article (10.1186/s13024-018-0288-y) contains supplementary material, which is available to authorized users.

## Background

Frontotemporal dementia (FTD) and amyotrophic lateral sclerosis (ALS) are two related neurodegenerative disorders with overlapping molecular disease pathways. In FTD, neuronal loss in the frontal and anterior temporal lobes gives rise to behavioral changes and/or language impairments [1]. In ALS, the degeneration of upper and lower motor neurons causes progressive muscle weakness limiting survival to 2–5 years after symptom onset [2]. TAR DNA binding protein 43 (TDP-43) has been identified as an important disease protein for both ALS and FTD, as most patients with ALS, and up to 50% of patients with FTD develop TDP-43 pathology [3]. While most ALS/FTD cases are sporadic, a familial component can be identified in approximately 10% and 40% of ALS and FTD patients, respectively [1, 2, 4]. Mutations in several genes can cause both ALS and FTD or ALS-FTD, including mutations in the gene encoding TDP-43 itself [5–7]. In FTD patients, TDP-43 pathology is frequently associated with progranulin haploinsufficiency caused by loss-of-function mutations in the *progranulin* (*GRN*) gene [8–10]. Such mutations, which result in ~ 50% reductions of progranulin protein (PGRN) levels [11–13], also rarely cause FTD-ALS [14] and genetic variations in *GRN* have been associated with the age of onset, disease duration and risk of disease in ALS [15].

The preferential expression of PGRN in neurons and activated microglia points towards its most important functions in the central nervous system [16, 17]. PGRN is an important modulator of neuroinflammation and of microglial recruitment and activation [18, 19], as PGRN shortage results in an exaggerated inflammatory response after brain insults [20, 21] and in an impaired microglial phagocytosis [22]. In addition, PGRN has neurotrophic effects which include stimulation of neurite outgrowth, of synaptic connectivity and of neuronal survival [11, 23–28]. At the subcellular level, PGRN ends up in late endosomes/lysosomes and facilitates the lysosomal clearance function [29], possibly by controlling the acidification of lysosomes [30] and by acting as a chaperone of degradation enzymes [31–33].

How shortage of PGRN causes TDP-43 pathology remains incompletely understood, but dysfunctional lysosomal degradation pathways with reduced clearance of TDP-43 could contribute to the accumulation of pathological TDP-43 species [34, 35].

We previously showed that PGRN has neuroprotective effects in a zebrafish model of mutant TDP-43 induced motor neuron damage [36]. However, its effect in rodent TDP-43 models remains unexplored. We therefore studied the therapeutic potential of PGRN in a mutant TDP-43(A315T) mouse model with a progressive motor phenotype [37, 38].

## Methods

### Mice

The TDP-43(A315T) and *GRN* overexpressing mice were bred in a C57BL/6 J background and maintained as previously described [38, 39] and all mice in the study were fed DietGel®boost (ClearH20, Maine, USA). This gel food contains all necessary nutrients, but is a soft, high calorie, easily digestible paste containing hardly any fibers. Female littermates were used for the experiments. All experiments were approved by the Ethical Committee of the KU Leuven (P148/2011).

### Gene expression analysis

For qRT-PCR analysis, first-strand cDNA was synthesized using SuperScript III (Invitrogen). PCR reactions were performed using TaqMan assays (Applied Biosystems, Foster City, CA, USA) for *Iba1*, *TARDBP*, *Tardbp* and *Grn*. Gene expression was normalized to the expression of three reference genes using SYBR Green reagents (Thermo Fisher Scientific) with the following primer pairs: adaptor-related protein complex 3, delta 1 subunit (*Ap3d1*) (forward, 5’-CAAGGGCAGTATCGACCGC-3′; reverse, 5’-GATCTCGTCAATGCACTGGGA-3′), MON2 homolog (*Mon2*) (forward, 5’-CTACAGTCCGACAGGTCGTGA-3′; reverse, 5’-CGGCACTGGAGGTTCTATATCTC-3′) and F-box protein 38 (*Fbxo38*) (forward, 5’-ATGGGACCACGAAAGAAAAGTG-3′; reverse, 5’-TAGCTTCCGAGAGAGGCATTC-3′). Expression levels were analyzed using qBase+ (v.3.0, Biogazelle, Zwijnaarde, Belgium).

RNA sequencing was performed by the Nucleomics Core Facility (VIB, Leuven, Belgium). From extracted RNA, libraries were made using the Illumina TruSeq Stranded mRNA Library protocol. These libraries were sequenced on an Illumina HiSeq single-end 51 bp and an average of 19.3 million reads per sample (range 17.7–21.2). To estimate the expression of the transcript of every sample, reads were counted using *Salmon (v0.8.1)* [40] against the Ensembl transcript for the mouse reference genome mm10. Gene expression as then estimate from the protein coding transcripts using the *tximport* function the R-package *tximport (v1.6.0)* [41]. Differential expression of coding genes was performed using the R-package *EdgeR (v3.20.5)* [42]. Genes were regarded as differentially expressed when the FDR-adjusted *p*-value (Benjamini and Hochberg method) was smaller than 0.05 and the absolute value of the log fold change (logFC) was larger or equal to 1. Genes were regarded as corrected by *GRN* overexpression, when the gene was differentially expressed between NTG and TDP-43(A315T) (FDR < 0.05 and |logFC| ≥ 1) mice but not differentially expressed between NTG and TDP-43(A315T)xGRN mice (unadjusted p-value ≥0.05). Pathway analysis were performed using Ingenuity Pathway Analysis (QIAGEN). Gene set enrichment analysis (GSEA) on gene ontology (GO) terms was performed using the logFC values of the differential expression analysis and the R-packages *gage (v2.28.0)* and *gageData_(v2.16.0).*

### Histology

Spinal cords from mice of 200–240 days were fixed overnight in 4% glutaraldehyde in PBS (pH 7.4), washed with PBS and post-fixed for 2 h in 1% osmium tetroxide. Dehydration of the samples was performed using an ethanol gradient as follows: 15 min in 50%, 15 min in 70%, 15 min in 90% and 3 times 15 min in 100% ethanol. After a washing step in 100% propylene oxide (Sigma-Aldrich, St. Louis, Missouri, USA), the samples were embedded using increasing ratios of TAAB medium (TAAB 812 Resin Premix kit 812, TAAB Laboratories, Aldermaston, Berkshire, United Kingdom) and propylene oxide: 1–2 h with a 1:2 ratio, overnight with a 1:1 ratio, 1–2 h with a 2:1 ratio and twice 2 h with 100% TAAB medium. The samples were subsequently baked for 72 h in a 60 °C oven before cutting 1 μm semi thin sections with a Reichert Ultracut microtome (Reichert, Wien, Austria). Sections were stained 20 s with toluidine blue. Photographs of the lateral column of the spinal cord were taken with a Zeiss Imager.M1, using a 100X objective. To measure the average area of all axon fibers, a custom analysis macro was created in ImageJ (v.1.49, NIH, Bethesda, MD, USA) which was then applied to each image.

### Western blot

Proteins were extracted from brain cortex samples using T-PER reagent (Sigma-Aldrich) supplemented with Complete™, EDTA-free protease inhibitor cocktail (Sigma-Aldrich). Protein concentrations were determined using the microBCA kit (Thermo Fisher Scientific) according to the manufacturer’s instructions. To study the insoluble protein fraction, equal amounts of protein extract were first centrifuged at 4 °C at maximum speed during 20 min. The obtained supernatant was used as the soluble fraction. The pellet was washed and gently vortexed with 1000 μl RIPA buffer (Sigma) containing cOmplete™ (Sigma), and PhosSTOP™ (Sigma). After a second centrifugation step the pellet was dissolved in sodium dodecyl sulfate (SDS) and used as the insoluble fraction.

Reducing sample buffer (Thermo Fisher Scientific) was added to samples containing equal amounts of protein and heated for 5 min at 95 °C before separation on a sodium dodecyl sulfate–polyacrylamide electrophoresis gel. After electrophoresis, the proteins were transferred to a polyvinylidene difluoride membrane (Merck Millipore, Darmstadt, Germany). Non-specific binding was blocked using 5% blotting-grade blocker (Bio-rad, Hercules, CA, USA), diluted in Tris-Buffered Saline Tween (50 mM TRIS, 150 mM NaCl, 0.1% Tween-20; Applichem, Darmstadt, Germany) for 1 h at room temperature before. Primary antibodies were incubated overnight at 4 °C, diluted in blocking-grade buffer and directed against TDP-43 (1:1000, ProteinTech, Chicago, IL, USA), GAPDH (1:2500, Thermo Fisher Scientific) PGRN (1:200 R&D systems) and CTSD (1:1000, Abcam). HRP-coupled secondary antibodies (1:5000, Dako, Agilent), diluted in TBS-T, were incubated for 1 h at room temperature. Blots were visualized using the enhanced chemiluminescent substrate (Thermo Fisher Scientific) and imaged with an ImageQuant LAS 4000 system (GE Healthcare, Uppsala, Sweden). Western blots were quantified using ImageQuant TL (v. 7.0). To quantify total TDP-43 levels, the sum of the intensities of the mTDP-43 band and the hTDP-43 band was used. CTSD activity measurements were performed using the CTSD activity assay kit (Abcam), following the manufacturer’s instructions.

### Statistical analyses

All statistical analyses were performed using GraphPad Prism (v 7.01, GraphPad Software, Inc., San Diego, CA, USA), unless otherwise stated. Statistical tests used are indicated in the figure legends. Non-parametric testing was performed when the data was not normally distributed.

## Results

### Progranulin overexpression reduces insoluble TDP-43 levels in TDP-43(A315T) mice

To study the therapeutic potential of human *GRN* overexpression on TDP-43(A315T) induced neurodegeneration, TDP-43(A315T) mice, which express human mutant TDP-43 under the control of the prion promoter, were crossed with human *GRN* overexpressing mice, which carry a copy of human *GRN* cDNA in the ROSA26 locus resulting in human PGRN protein overexpression [39]. TDP-43(A315T) mice develop a progressive motor phenotype when sudden death due to intestinal obstructions is prevented by putting them on a diet only consisting of an easy to digest nutrient gel [37, 38].

**Fig. 1:**
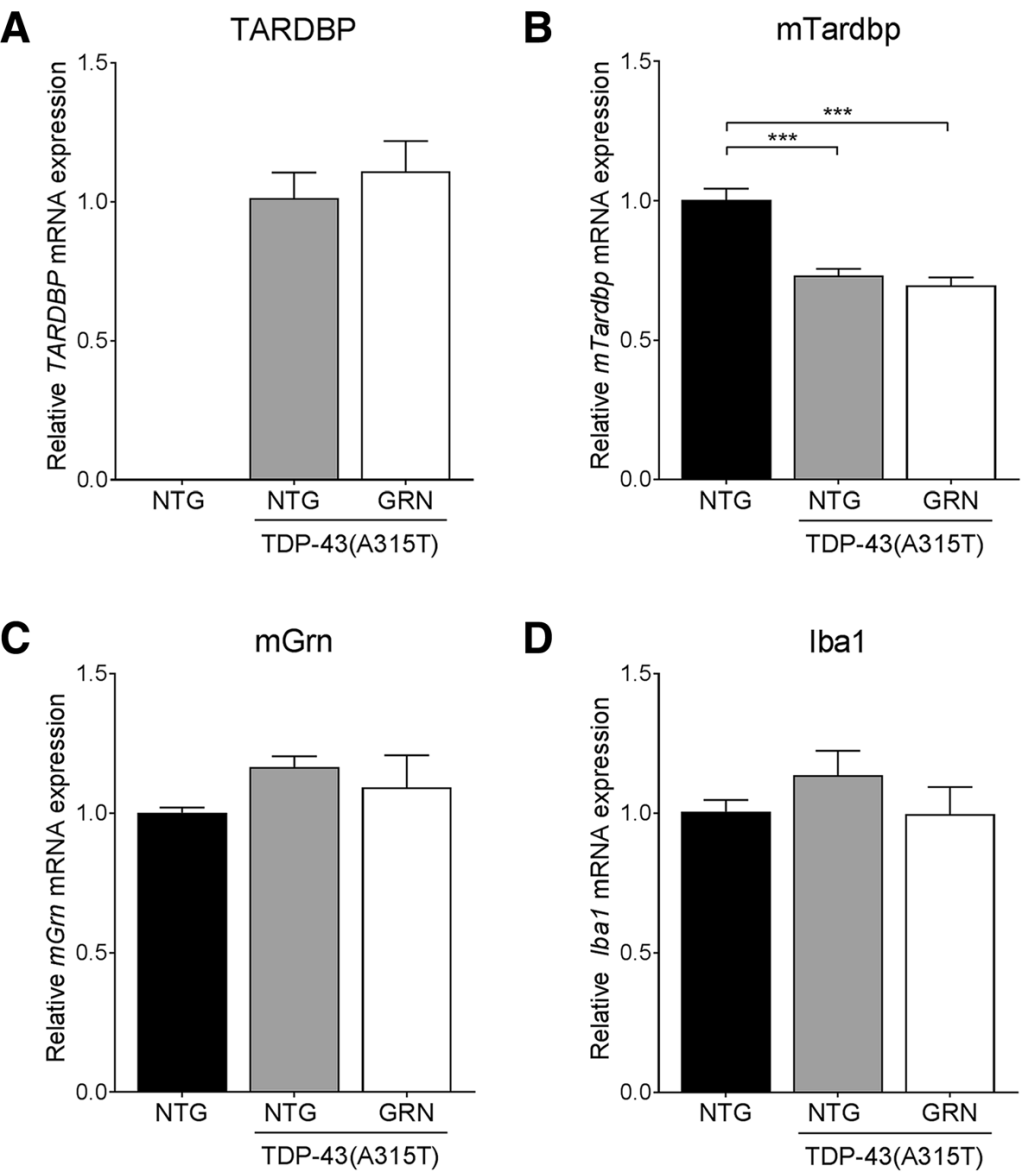
PGRN overexpression has no effect on TDP-43 RNA levels. RNA expression of the human *TDP-43(A315T)* transgene (**a**), mouse *Tardbp* (**b**) and mouse *Grn* (**c**) in the spinal cord is unchanged by PGRN overexpression. ***p < 0.001, one-way ANOVA. **d** No significant changes were observed in *Iba1* expression in the spinal cord. Data are shown as mean ± SEM

**Fig. 2:**
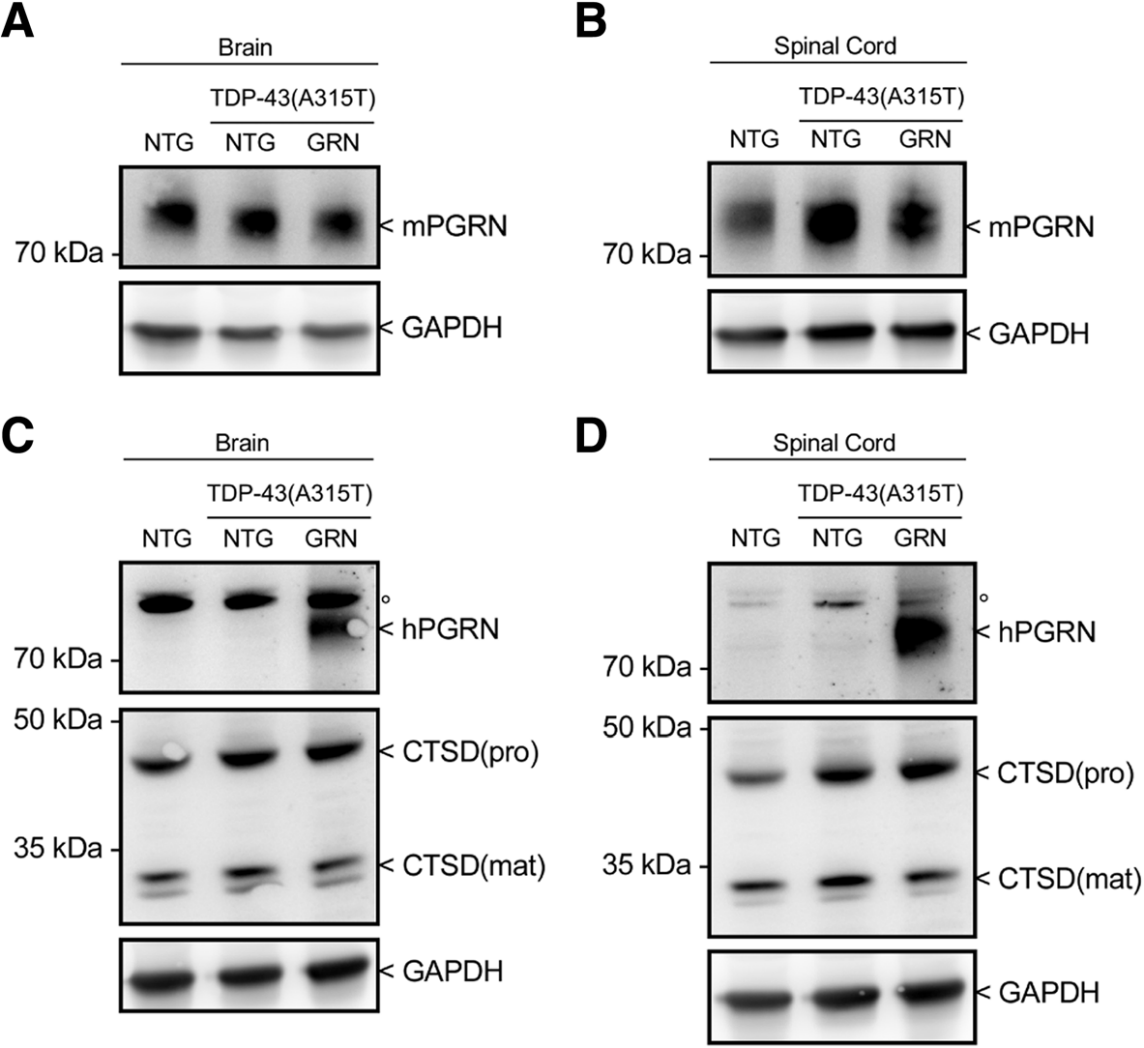
Human PGRN overexpression has no effect on mouse PGRN or CTSD protein levels. **a-b** Protein expression of mouse PGRN in brain and spinal cord from NTG, TDP-43(A315T) and TDP-43(A315T)xGRN mice. **c-d** Protein expression of human PGRN and mouse CTSD in brain and spinal cord NTG, TDP-43(A315T) and TDP-43(A315T)xGRN mice. °Aspecific band

Overexpression of PGRN had no effect on the TDP-43(A315T) transgene expression (Fig. 1a) and did not affect the downregulation of endogenous TDP-43 by the human TDP-43 transgene (Fig. 1b and Additional file 1: Figure S2A). No significant changes in mouse *Grn* and the microglial marker *Iba1* were observed in TDP-43(A315T) mice and PGRN overexpression did not influence the expression of these genes (Fig. 1c-d). Overexpression of PGRN also left the levels of endogenous PGRN unaltered (Fig. 2a-b and Additional file 1: Figure S1A-B). Shortage of PGRN has previously been shown to impair lysosomal function and to induce lysosomal enzymes, such as cathespin D (CTSD) [31, 34]. Overexpression of PGRN did not result in altered CTSD levels (Fig. 2c-d and Additional file 1: Figure S1C-D) or a change in CTSD activity (Additional file 1: Figure S1E-H).

**Fig. 3:**
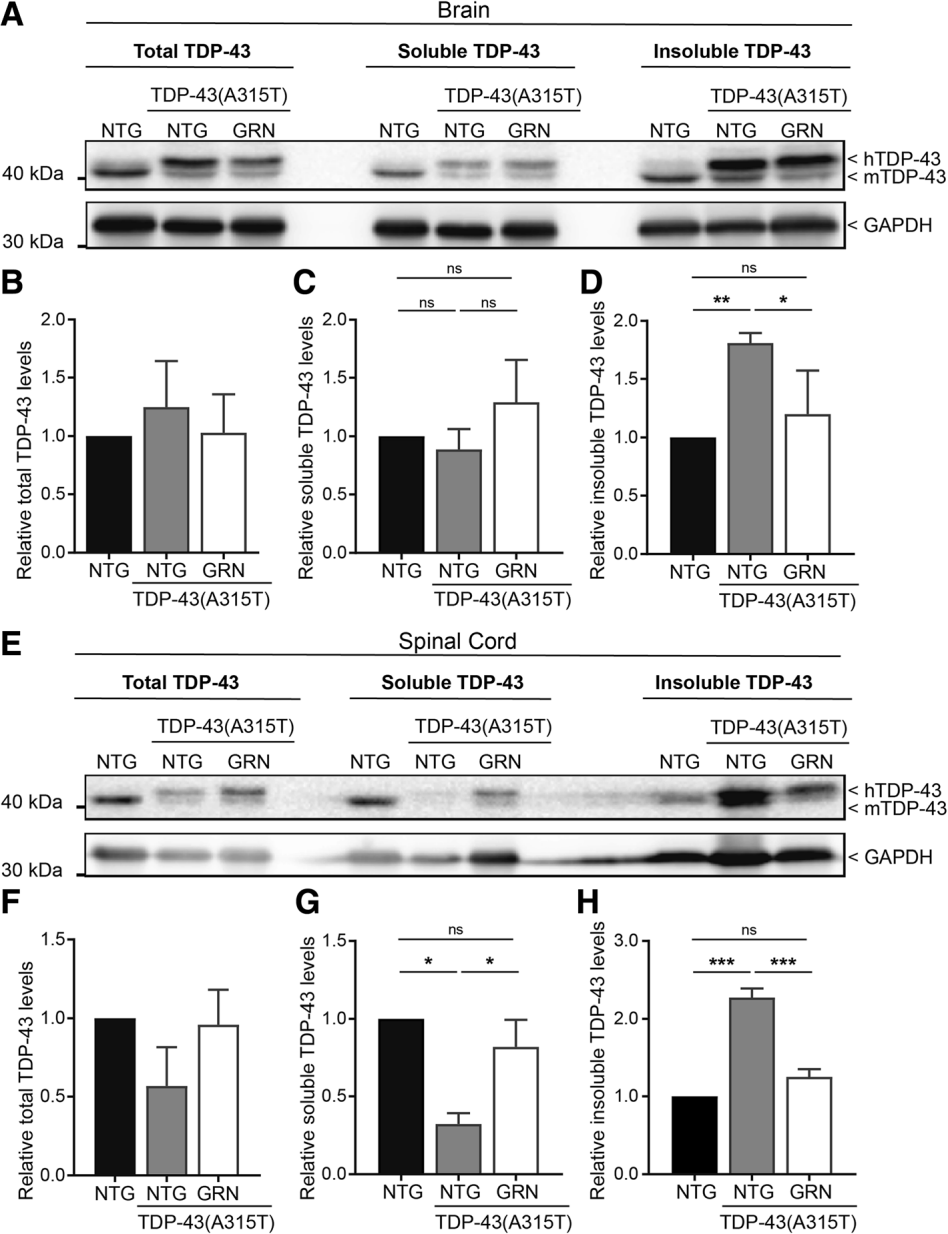
PGRN overexpression reduces insoluble TDP-43 levels. **a, e** Western blot analysis of total, soluble and insoluble TDP-43 in brain (**a**) and spinal cord (**e**) from NTG, TDP-43(A315T) and TDP-43(A315T)xGRN mice. Quantification of blots (**b-d** and **f-h**) are shown as mean ± SEM (*n* = 3 per group, * *p* < 0.05, ** *p* < 0.001, *** *p* < 0.0001, Tukey-Kramer multiple comparison test)

TDP-43(A315T) mice do not display clear mislocalisation of TDP-43 or TDP-43 inclusions. However, accumulation of TDP-43 in the insoluble fraction was observed in TDP-43(A315T) mice, especially in the spinal cord (Fig. 3). Interestingly, overexpression of PGRN reduced this insoluble fraction of TDP-43, most efficiently in the spinal cord. This suggests that PGRN can reduce the formation or enhance the clearance of insoluble TDP-43 species.

### Degeneration of large axon fibers in the lateral spinal cord is prevented by progranulin overexpression

**Fig. 4:**
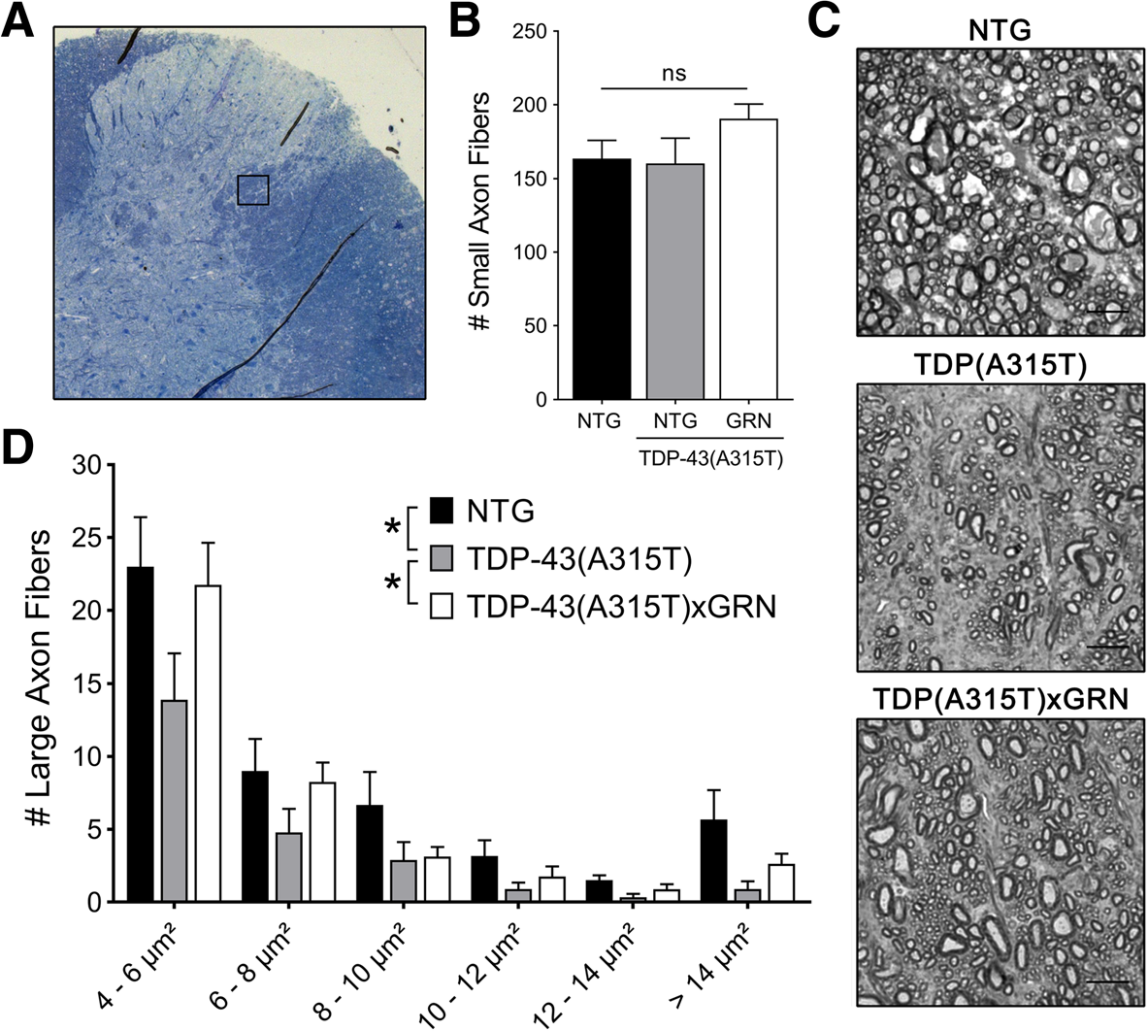
PGRN overexpression prevents degeneration of large axon fibers. **a** Schematic overview of the region in the lateral spinal cord used for the analysis of axon fibers as indicated by the black square. **b** No difference was found in the number of small axon fibers (1–4 μm^**2**^) in this region. **c** Representative images of the axon fibers in the lateral spinal cord (Scale bar = 10 μm). **d** The mean number of large axon fibers in NTG and TDP-43(A315T)xGRN mice was significantly higher across all size groups, compared to TDP-43(A315T) mice (*n* = 6–9 per group, *p < 0.05, Wilcoxon signed-rank test). Data are shown as mean ± SEM

A prominent loss of spinal motor neuron cell bodies is lacking in TDP-43(A315T) mice [38]. The most dramatic pathological hallmark of this mouse model is the loss of large axon fibers in the lateral column of the spinal cord [37, 38]. The number of small axon fibers (1–4 μm^2^) in this region was not affected by TDP-43(A315T) expression and was also not changed by PGRN overexpression (Fig. 4a-b). However, the overall number of large axon fibers was significantly reduced in TDP-43(A315T) mice and the overexpression of PGRN mitigated this phenotype (Fig. 4c).

### Progranulin overexpression extends disease duration in TDP-43(A315T) mice

**Fig. 5:**
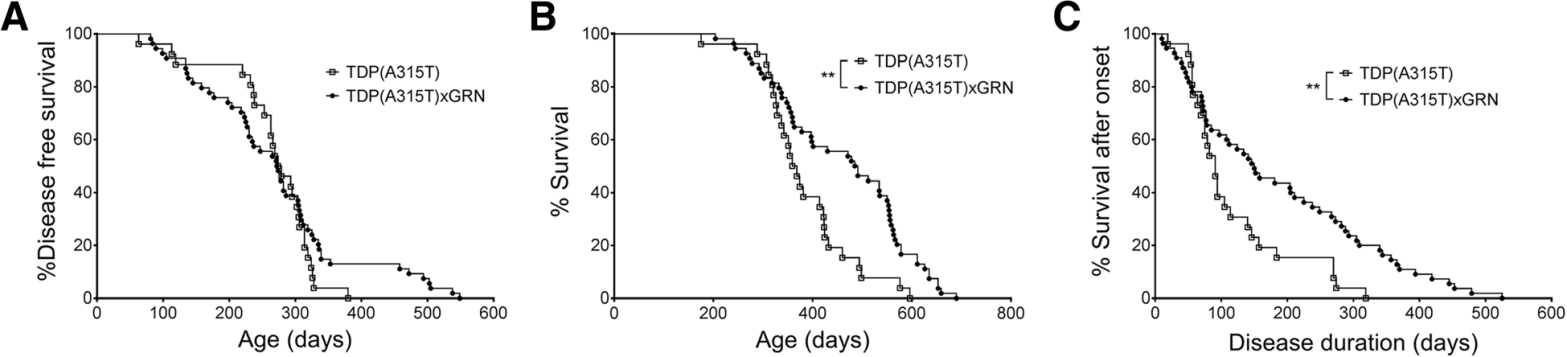
PGRN overexpression increases survival and disease duration in TDP-43(A315T) mice. **a** Disease onset is not affected by PGRN overexpression (*n* = 26 and 54 for TDP-43(A315T) and TDP-43(A315T)xGRN mice, respectively). **b** The survival is significantly increased by PGRN overexpression. Median survival: 364 d (TDP-43(A315T)) versus 491 d (TDP-43(A315T)xGRN). **p < 0.01, Log-rank test. **c** PGRN overexpression significantly increased the survival after symptom onset. Median disease duration: 91 d (TDP-43(A315T)) versus 150 d (TDP-43(A315T)xGRN). **p < 0.01, Log-rank test

Next, the effect of PGRN was assessed on the clinical phenotype of TDP-435A315T) mice. The disease onset, as determined by the appearance of a swimming gait and the inability to lift up the lower part of the body from the ground, which is quite variable in this model, was not altered by PGRN overexpression (Fig. 5a). In contrast, PGRN overexpression did significantly increase the survival of TDP-43(A315T) mice (Fig. 5b). Also the disease duration after onset, was clearly prolonged by the overexpression of PGRN (Fig. 5c).

With these results, we provide evidence for a protective effect of PGRN on TDP-43(A315T) induced neurodegeneration, as the increase in disease duration was accompanied by reduced insoluble TDP-43 levels and protection of large axon fibers in the spinal cord.

### Transcriptome analysis does not reveal a single pathway underlying PGRN neuroprotection

To further characterize the neuroprotective effects of PGRN, we performed a transcriptome analysis on the spinal cord from 50-day-old NTG, TDP-43(A315T) and TDP-43(A315T)xGRN mice (*n* = 3 per group). The downregulation of endogenous mouse *Tardbp* in TDP-43(A315T) mice was confirmed and this was not altered by PGRN overexpression (Additional file 1: Figure S2A). The expression of total *GRN* RNA in the TDP-43(A315T)xGRN mice compared to endogenous mouse *Grn* was estimated to be 2.36 (95% CI 1.00–3.72) (Additional file 1: Figure S2A). The samples clustered according to genotype (Additional file 1: Figure S2B) and a gene set enrichment analysis revealed especially reduced expression in ribosomal and mitochondrial genes in TDP-43(A315T) mice compared to NTG controls (Additional file 1: Table S1). PGRN overexpression particularly influenced extracellular matrix genes, but did not correct the reduced expression in ribosomal and mitochondrial genes. No changes in microglial or lysosomal genes were observed. There was a differential gene expression in 35 genes when comparing TDP-43(A315T) to NTG controls (Additional file 1: Table S2). For 7 of those genes, the expression was corrected by PGRN overexpression (Additional file 1: Figure S2C). A pathway analysis of these genes did not provide evidence for a protective effect of PGRN on one specific pathway, it rather hinted at a pleiotropic effect of PGRN on different pathways, some with possible importance for ALS-FTD (*Rsad2* is an ER stress-induced protein involved in cell defense, *Top2a* is a topoisomerase involved in replication- and transcription-associated DNA breaks and repair, *Fbxo22* is part of the ubiquitin ligase complex involved in ubiquitination and degradation of proteins). Interestingly, using String DB, the proteins encoded by these 3 genes clustered in a protein-protein interaction network (Additional file 1: Figure S2D). The effect of PGRN on the expression of the key player, Rsad2, was confirmed using Western blot (Additional file 1: Figure S3). Although little is known about the concert function of these gene products, both *Rsad2* and *Fbxo22* are involved in the innate and adaptive immune system in the central nervous system and *Top2* is involved in inflammation-induced DNA damage [43, 44].

## Discussion

Neuronal TDP-43 positive inclusions are the main pathological hallmark in most ALS patients and in about half of FTD patients and the degree of TDP-43 pathology correlates with the degree of neuronal loss [45]. In this study, we investigated the possibility of a therapeutic effect of PGRN on the disease progression of an ALS mouse model with mutant TDP-43(A315T) overexpression. By crossbreeding human PGRN overexpression mice to TDP-43(A315T) mice, we could indeed observe a significant improvement in the survival of these mutant TDP-43 mice. PGRN reduced the levels of insoluble TDP-43 and protected large axon fibers in the lateral column of the spinal cord of these animals. This suggests that PGRN has protective effects by lessening the production or enhancing the clearance of insoluble TDP-43 species. A transcriptome analysis did not provide evidence for a neuroprotective effect of PGRN through modulation of microglial activation or lysosomal function, two processes known to be dependent on PGRN [46]. On the contrary, we recently described a neurotrophic effect of PGRN after nerve crush which could be linked to lysosomal effects of PGRN as chaperone of the lysosomal protease CTSD or cathepsin D [31]. CTSD was identified using a similar design analysis in which we looked for genes with differential expression which had corrected expression levels after PGRN treatment. However, these experiments were performed in Grn-deficient mice, with known defects in lysosomal function. In this study, the TDP-43(A315T) mice had normal baseline PGRN levels. Although our transcriptome analysis did not point towards lysosomal function, there is some evidence that TDP-43 pathology could potentially be a direct consequence of defective autophagy and lysosomal function: inhibition of lysosomes resulted in the redistribution of TDP-43 protein to the cytoplasm [47] and PGRN was shown to be necessary to maintain the autophagic flux to prevent the accumulation of pathological forms of TDP-43 [35].

How PGRN specifically reduces insoluble TDP-43 levels and prevents TDP-43 mediated neurodegeneration requires further investigation. Of the 35 genes with differential expression in the mutant TDP-43 mice, PGRN corrected the levels of 7 of them. Some of these are indirectly connected to each other in a protein interaction network and can be linked to innate or adaptive immunity. *Radical S-Adenosyl Methionine Domain containing 2* (*Rsad2*) is an interferon-inducible ER protein that functions as an innate immunity factor [43] and is also upregulated in mutant *PINK1* fibroblasts [48]. Finally, *Fbxo22* is a member of the ubiquitin ligase complex important for ubiquitination and degradation of proteins and also plays a role in macrophage activation [49]. *Topoisomerase II alfa* (*Top2a*) catalyzes transient breaking and ligation of dsDNA during transcription and translation and has been involved in inflammation-induced DNA damage [44]. Apart from effects on neuroinflammation, effects on DNA damage, which is an emerging disease mechanism in ALS-FTD [50], could be of interest.*Top2a* and *GRN* are upregulated in glioblastomas [51, 52] and *GRN* overexpression was shown to protect against DNA damage induced by the Top2a inhibitor tomozolomide in glioblastoma cell lines [52].

In addition, a direct neurotrophic effect of PGRN may occur without changes in gene expression. Neuroprotective effects of PGRN have also been observed in models of other brain disorders, suggesting that PGRN may have beneficial effects in various brain diseases not restricted to FTD with *GRN* haploinsufficiency [53]. PGRN treatment was shown to be therapeutic in mouse models for Parkinson’s disease [54, 55], Alzheimer’s disease [22] and stroke [56, 57] and the neuroprotective effects were mostly attributed to modulation of neuroinflammation, effects.

## Conclusion

Our data show that PGRN reduces insoluble TDP-43 levels, slows down axonal degeneration and prolongs survival in mutant TDP-43 mice. With our study, we add TDP-43 linked neurodegeneration to the list of disorders for which PGRN treatment could be beneficial.

## Additional file

Additional file 1: **Table S1.** Results of the gene set enrichment analysis. **Table S2**. List of the 35 differentially expressed genes when comparing NTG controls to TDP-43(A315T) mice. **Figure S1.** PGRN overexpression does not affect endogenous PGRN or CTSD levels/activity. **Figure S2.** RNAseq results. **Figure S3.** Western blot of Rsad2. (A) Western blot for Rsad2 in brain and spinal cord lysates from NTG, TDP-43(A315T) and TDP-43(A315T)xGRN mice. (B) Quantification of Rsad2 bands from brain and spinal cord of NTG, TDP-43(A315T) and TDP-43(A315T)xGRN mice (*n* = 3 per group, * *p* < 0.05, Tukey-Kramer multiple comparison test). (ZIP 1920 kb)

## Abbreviation

ALS: Amyotrophic lateral sclerosis
EDTA: Ethylenediaminetetraacetic acid
FDR: False discovery rate
FTD: Frontotemporal dementia
GO: Gene ontology
*GRN*: (Progranulin gene)
GSEA: Gene set enrichment analysis
logFC: Log fold change
PBS: Phosphate buffered saline
PGRN: (Progranulin protein)
qRT PCR: Quantitative reverse transcriptase polymerase chain reaction
TDP-43: TAR DNA binding protein 43
T-PER: Tissue Protein Extraction Reagent
