# Supplementary material for: Progranulin reduces insoluble TDP-43 levels, slows down axonal degeneration and prolongs survival in mutant TDP-43 mice
Source: Mol Neurodegener. 2018 Oct 16;13:55. doi: 10.1186/s13024-018-0288-y (PMC6192075; doi:10.1186/s13024-018-0288-y)
Supplement: Supplementary file 1 — Table S1. Results of the gene set enrichment analysis. Table S2. List of the 35 differentially expressed genes when comparing NTG controls to TDP-43(A315T) mice. Figure S1. PGRN overexpression does not affect endogenous PGRN or CTSD levels/activity. Figure S2. RNAseq results. Figure S3. Western blot of Rsad2. (A) Western blot for Rsad2 in brain and spinal cord lysates from NTG, TDP-43(A315T) and TDP-43(A315T)xGRN mice. (B) Quantification of Rsad2 bands from brain and spinal cord of NTG, TDP-43(A315T) and TDP-43(A315T)xGRN mice (n = 3 per group, * p < 0.05, Tukey-Kramer multiple comparison test). (ZIP 1920 kb) [file 13024_2018_288_MOESM1_ESM.zip › Supplementary Materials.docx]

Supplementary Materials

**Table S1: Results of the gene set enrichment analysis.** Top gene ontology-terms with lower expression in TDP-43(A315T) mice compared to NTG mice.

**Table S2**: List of the 35 differentially expressed genes when comparing NTG controls to TDP-43(A315T) mice. The table also contains the results of the differential expression analysis for these 35 genes for the comparison NTG controls to TDP-43(A315T)xGRN and TDP-43(A315T) controls to TDP-43(A315T)xGRN. logFC: log fold change, the log2 increase or decrease in expression compared to NTG. logCPM: log count per millions, the log2 of the overall expression over both compared groups expressed in count per millions (mapped reads counts scaled by the total number of reads sequenced times one million). LR: Likelihood ratio. P values: p-value calculated from the LR. FDR: false discovery rate corrected P value by the Benjamini and Hochberg method.

Figure S1: PGRN overexpression does not affect endogenous PGRN or CTSD levels/activity. Quantification of blots from brain and spinal cord lysates from NTG, TDP-43(A315T) and TDP-43(A315T)xGRN mice for endogenous PGRN (A-B) and CTSD (C-D); data are shown as mean ± SEM (n = 3 per group, p = 0.13, 0.71, 0.71 (PRO) and 0.55 (MAT), 0.18 (PRO) and 0.14 (MAT), for A-D, respectively). (E-F) CTSD activity in brain and spinal cord lysates from NTG, TDP-43(A315T) and TDP-43(A315T)xGRN mice (n = 3 per group, p = 0.09 and 0.17, for E-F, respectively).

**Figure S2: RNAseq results. (**A) The expression of mouse *Tardbp* (m*Tardbp*), human *TARDBP*, mouse *Grn* (m*Grn*) and human *GRN* in NTG, TDP-43(A315T) and TDP-43(A315T)xGRN mice, depicted in black, grey and white respectively. ***FDR adjusted p<0.001, edgeR. mRNA expression expressed in rkpm (reads per kilobase of exon per million reads mapped). Data is shown as mean ± SEM. (B) Heat map of differentially expressed gens as assessed by EdgeR. Cutoffs FDR adjusted p-values<0.05 |logFC|>1. Rows and columns were subject to unsupervised hierarchical clustering as shown by the dendrograms on the left side and top of the heatmap, showing a clear separation between the different mouse models. The columns, representing the different mice, are labeled black, grey and white for the NTG, TDP-43(A315T) and TDP-43(A315T)xGRN mice respectively. In the heat map, red indicates higher expression values and green indicates lower expression values. For every row the gene expression is converted into z-score, showing the deviation from the mean expression of that gene over the different mice. (C) The expression of the 7 genes, for whom the expression was corrected by h*GRN* overexpression, in NTG, TDP-43(A315T) and TDP-43(A315T)xGRN mice, depicted in black, grey and white respectively. Data are shown as mean ± SEM. (D) STRING Interaction Network, showing the proteins encoded by the 7 genes corrected by h*GRN* overexpression. Two major clusters were found: the red cluster is associated with cell cycle processes and the yellow cluster is associated with defense response. Colored proteins are input proteins, while gray proteins are secondary proteins that interact with the input proteins. Figure made by String DB web interface (https://string-db.org) using the human orthologues of the mouse genes, clustered with MCL clustering and inflation parameter set to 3.

**Figure S3**: **Western blot of Rsad2**. (A) Western blot for Rsad2 in brain and spinal cord lysates from NTG, TDP-43(A315T) and TDP-43(A315T)xGRN mice. (B) Quantification of Rsad2 bands from brain and spinal cord of NTG, TDP-43(A315T) and TDP-43(A315T)xGRN mice (n = 3 per group, * p<0.05, Tukey-Kramer multiple comparison test).
