# Supplementary figures and images for: Progranulin reduces insoluble TDP-43 levels, slows down axonal degeneration and prolongs survival in mutant TDP-43 mice
Source: Mol Neurodegener. 2018 Oct 16;13:55. doi: 10.1186/s13024-018-0288-y (PMC6192075; doi:10.1186/s13024-018-0288-y)

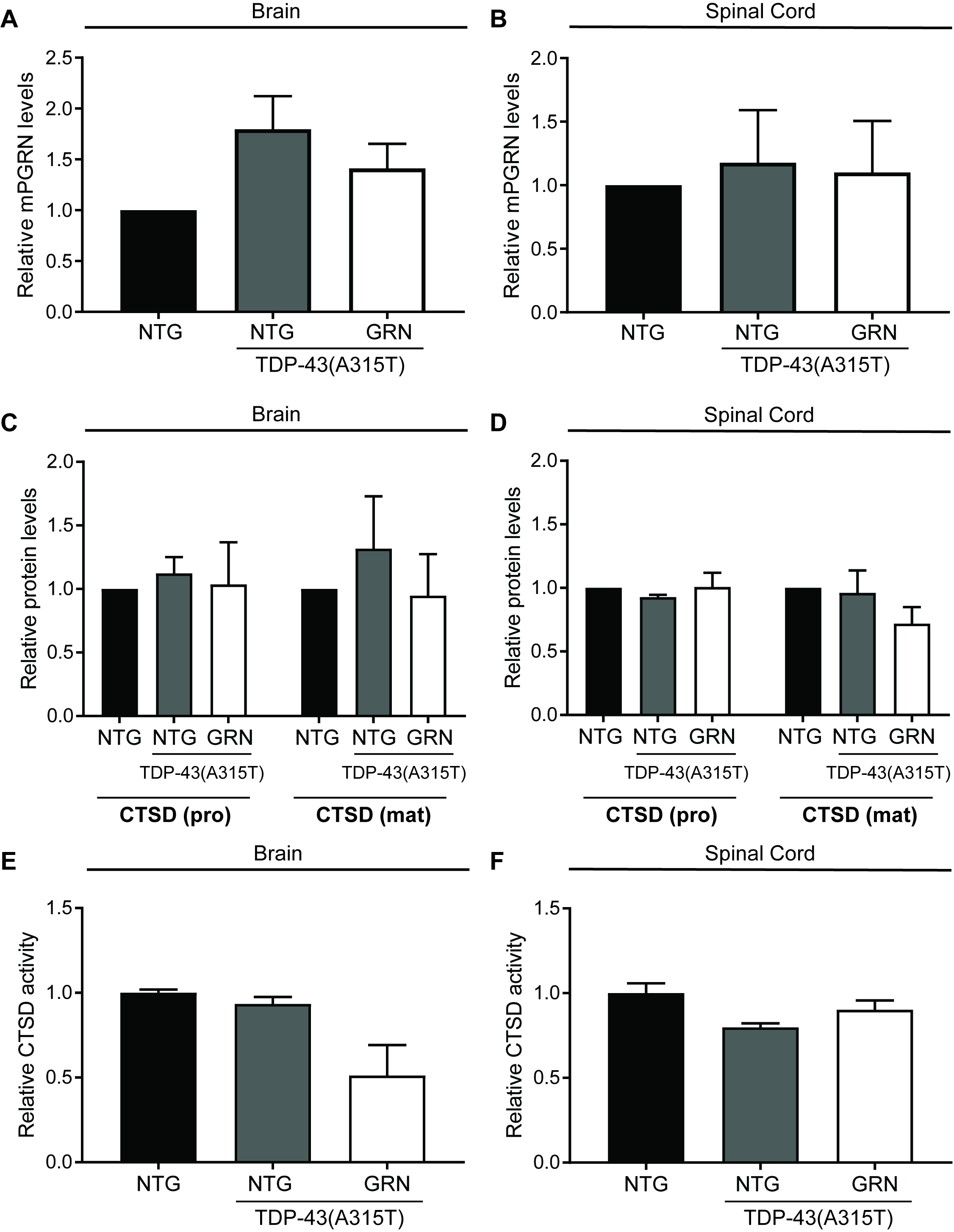

Supplement: Supplementary file 1 — Table S1. Results of the gene set enrichment analysis. Table S2. List of the 35 differentially expressed genes when comparing NTG controls to TDP-43(A315T) mice. Figure S1. PGRN overexpression does not affect endogenous PGRN or CTSD levels/activity. Figure S2. RNAseq results. Figure S3. Western blot of Rsad2. (A) Western blot for Rsad2 in brain and spinal cord lysates from NTG, TDP-43(A315T) and TDP-43(A315T)xGRN mice. (B) Quantification of Rsad2 bands from brain and spinal cord of NTG, TDP-43(A315T) and TDP-43(A315T)xGRN mice (n = 3 per group, * p < 0.05, Tukey-Kramer multiple comparison test). (ZIP 1920 kb) [file 13024_2018_288_MOESM1_ESM.zip › Supplementary Figure 1.tif]

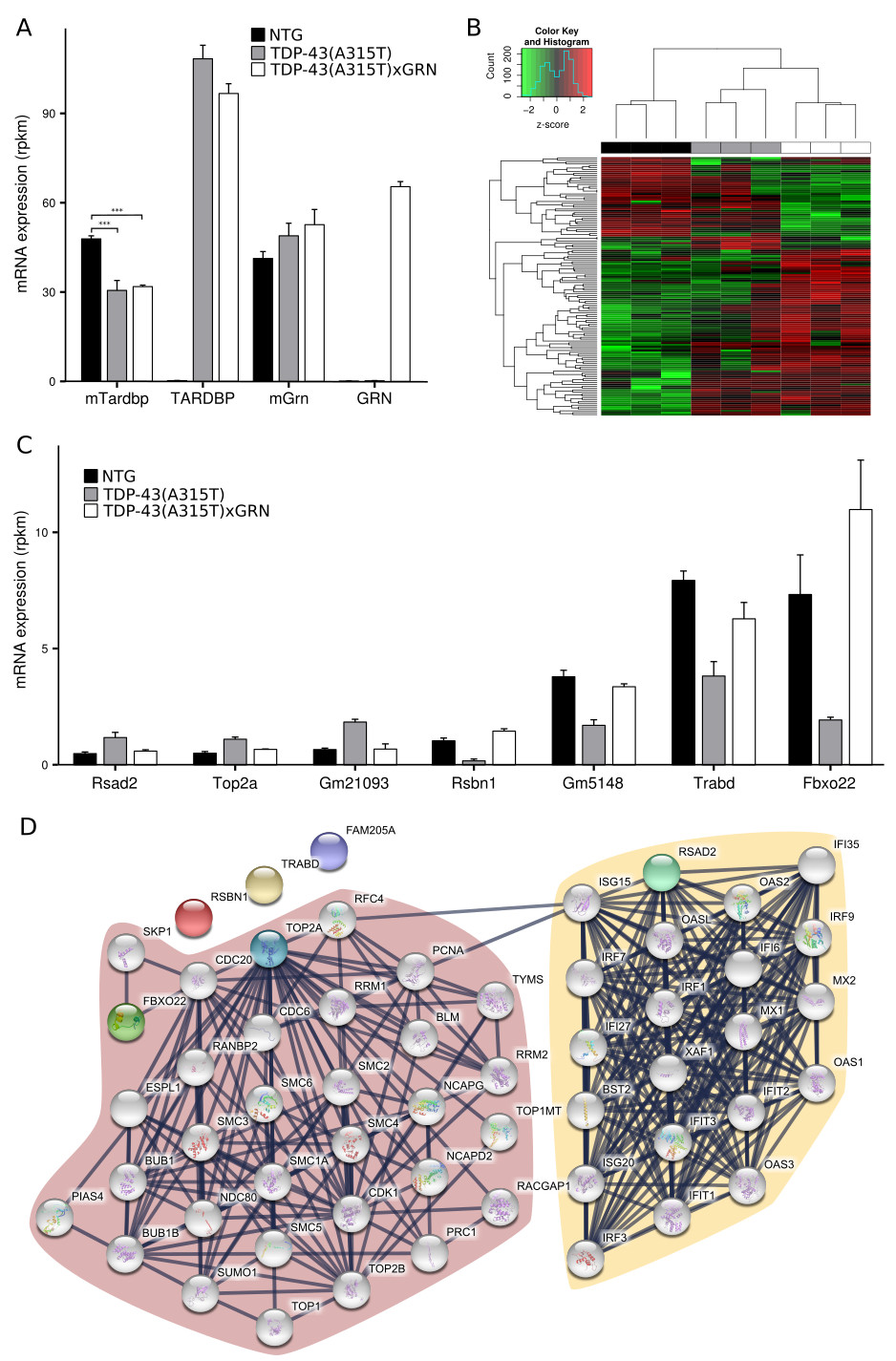

Supplement: Supplementary file 1 — Table S1. Results of the gene set enrichment analysis. Table S2. List of the 35 differentially expressed genes when comparing NTG controls to TDP-43(A315T) mice. Figure S1. PGRN overexpression does not affect endogenous PGRN or CTSD levels/activity. Figure S2. RNAseq results. Figure S3. Western blot of Rsad2. (A) Western blot for Rsad2 in brain and spinal cord lysates from NTG, TDP-43(A315T) and TDP-43(A315T)xGRN mice. (B) Quantification of Rsad2 bands from brain and spinal cord of NTG, TDP-43(A315T) and TDP-43(A315T)xGRN mice (n = 3 per group, * p < 0.05, Tukey-Kramer multiple comparison test). (ZIP 1920 kb) [file 13024_2018_288_MOESM1_ESM.zip › Supplementary figure 2.jpg]

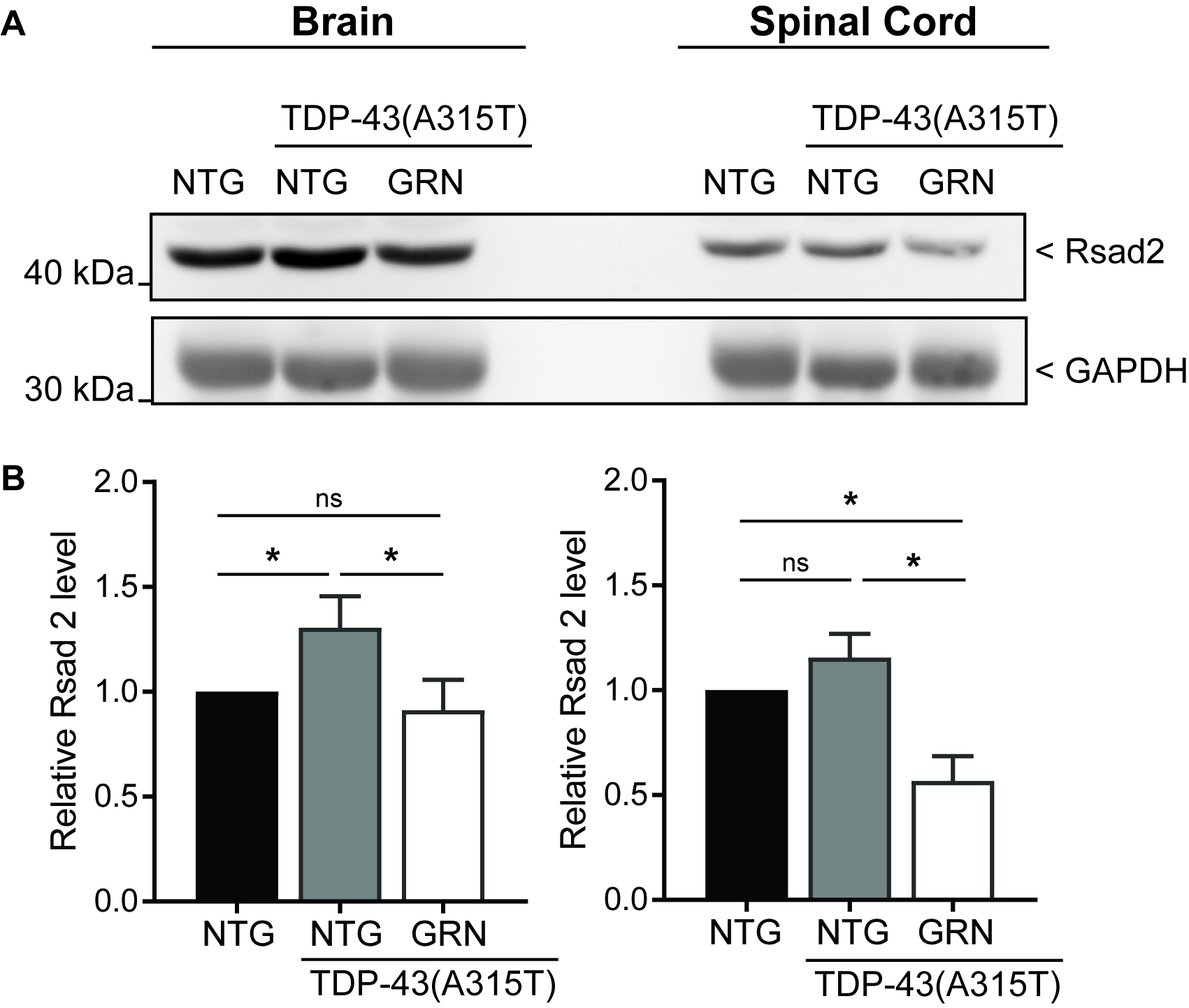

Supplement: Supplementary file 1 — Table S1. Results of the gene set enrichment analysis. Table S2. List of the 35 differentially expressed genes when comparing NTG controls to TDP-43(A315T) mice. Figure S1. PGRN overexpression does not affect endogenous PGRN or CTSD levels/activity. Figure S2. RNAseq results. Figure S3. Western blot of Rsad2. (A) Western blot for Rsad2 in brain and spinal cord lysates from NTG, TDP-43(A315T) and TDP-43(A315T)xGRN mice. (B) Quantification of Rsad2 bands from brain and spinal cord of NTG, TDP-43(A315T) and TDP-43(A315T)xGRN mice (n = 3 per group, * p < 0.05, Tukey-Kramer multiple comparison test). (ZIP 1920 kb) [file 13024_2018_288_MOESM1_ESM.zip › Supplmentary Figure 3.tif]
